# Prison primary care and non-communicable diseases: a data-linkage survey of prevalence and associated risk factors

**DOI:** 10.3399/bjgpopen19X101643

**Published:** 2019-05-29

**Authors:** Nat MJ Wright, Philippa Hearty, Victoria Allgar

**Affiliations:** 1 Clinical Research Director, Spectrum Community Health CIC, Wakefield, UK; 2 Research Assistant, Spectrum Community Health CIC, Wakefield, UK; 3 Reader in Medical Statistics, Hull York Medical School / Department of Health Sciences, University of York, York, UK

**Keywords:** prevalence, chronic conditions, prisoners, general practice, primary care

## Abstract

**Background:**

The size and mean age of the prison population has increased rapidly in recent years. Prisoners are a vulnerable group who, compared with the general population, experience poorer health outcomes. However, there is a dearth of research quantifying the prevalence of non-communicable diseases (NCDs) among prisoner populations.

**Aim:**

To explore both the prevalence of NCDs and their risk factors.

**Design & setting:**

A cross-sectional survey was undertaken that was compared with clinical records in two male prisons in the north of England.

**Method:**

Self-report surveys were completed by 199 prisoners to assess sociodemographic characteristics, general health, NCD prevalence, and risk factor prevalence. Data were checked against that retrieved from prison clinical records.

**Results:**

It was found that 46% reported at least one NCD and 26% reported at least one physical health NCD. The most common self-reported NCD was 'anxiety and depression' (34%), followed by 'respiratory disease' (17%), and 'hypertension' (10%). Having a physical health NCD was independently associated with increasing age or drug dependence.

The level of agreement between clinical records and self-report ranged from 'fair' for alcohol dependence (kappa 0.38; *P*<0.001) to 'very good' for diabetes (kappa 0.86; *P*<0.001).

**Conclusion:**

Compared with mainstream populations and despite high prevalence of risk factors for NCDs physical illness NCDs, with the exception of respiratory disease, are less common. However, poor mental health is more common. These differences are possibly owing to the younger average age of prison populations, since prevalence of risk factors was reported as high.

Secondary data analysis of clinical records is a more methodologically robust way of monitoring trends in prisoner population disease prevalence.

## How this fits in

Anecdotally, prisoners have a high prevalence of modifiable risk factors for NCDs. However, there is a paucity of international prevalence studies. This data linkage study of both self-report and primary care records highlights both disease and risk factor prevalence in this vulnerable group, which place a high burden of care on primary care services.

## Introduction

There are approximately 10.35 million prisoners worldwide, a population that has grown by almost 20% since 2000.^1^ At the time of writing, England and Wales has a prison population of 85 994, of which >95% are male.^2^ Alongside this growth in prisoner numbers worldwide, there has also been a steady increase in the number of older people in prison.^3^ Statistics have shown that in England and Wales, the number of prisoners aged ≥60 years has increased threefold between 1990–2000,^4^ and this is projected to further increase by 2020.^5^ As the prisoner population continues to grow, alongside the increase in the number of older prisoners, it is important to gain accurate estimates of the prevalence of chronic conditions among the prisoner population to ensure health services within prisons are meeting the health needs of their patients. Although the elevated prevalence of chronic infectious diseases in prisons is well documented,^6,7^ there is a paucity of research regarding the prevalence of chronic NCDs among prisoners, particularly in the English and Welsh prison setting.

The four primary NCDs, as identified by the World Health Organization (WHO), are cardiovascular disease, diabetes, cancers, and chronic respiratory diseases.^8^ These chronic NCDs have now become the leading cause of death worldwide and a major public health issue,^8^ with 38 million deaths per year suggested to be attributable to these diseases.^9^ In the UK, recent estimates indicated that approximately 89% of the 557 000 deaths in 2012 were accounted for by NCDs.^10^ Although these diseases affect individuals from all social backgrounds, there are clear inequalities in the burden of NCDs, with those vulnerable and from lower socioeconomic status disproportionately affected.^8,11^

Since prisoners are overrepresented in the most disadvantaged sectors of society,^11,12^ it is plausible that the incidence of NCDs is higher than in the general population. Empirical research conducted internationally would appear to suggest that such may be the case for some NCDs, in particular respiratory diseases.^13–16^

In the UK, incidence or prevalence studies on NCDs among the prisoner population are lacking, with the current evidence appearing to be limited to local descriptive health needs assessments.^17,18^ Therefore, in light of the paucity of empirical evidence pertaining to the prevalence of such conditions in this vulnerable group, a questionnaire of prisoners’ self-reported NCDs was undertaken, and cross-checked with a review of the primary care records to assess level of agreement between the two data sources.

The purpose of the study was to determine the prevalence of selected NCDs (hypertension, heart disease, stroke, cancer, respiratory diseases, diabetes, and anxiety and depression) and their risk factors (drug dependence, alcohol dependence, smoking, physical activity and diet) among prisoners from two prisons in the north of England.

## Method

The study took place in two prisons in the north of England: one medium-secure and one low-secure prison, with a total operational capacity of 2000 places. Following successful ethics and governance applications, questionnaire completion and data extraction from clinical records took place between October 2014–September 2015. At each of the prisons, 10% of the prisoner population was randomly selected by means of a computerised random number generator. Exclusion criteria were those deemed to pose a security threat to the researcher and those who lacked the mental capacity to provide consent. Of those invited, 199 participants provided written consent and were recruited; 120 from the medium-secure and 79 from the low-secure prison. Five prisoners declined to participate; in such cases, potential participants from the reserve list were recruited.

All consenting participants completed an edited version of the Health Survey for England 2007^19^ and the RAND 36-Item Health Survey.^20^ Data were collected pertaining to sociodemographic details, prison history, mental health history, general health, prevalence of NCDs, past and/or current smoking levels, past and/or current physical activity levels, and past and/or current diet and nutrition. Owing to the literacy difficulties faced by many prisoners,^21^ completion was facilitated through face-to-face support from the researcher. Participants’ height and weight were also measured.

Medical records for each consenting participant were also reviewed to check for agreement between the self-reported prevalence of an NCD and the diagnosis held on participants’ clinical records. To assure quality, a random 10% sample of the retrieved clinical records was checked by a senior GP and any discrepancies resolved through discussion with the researcher.

Data were summarised using mean (standard deviation [SD]), median (interquartile range [IQR]), or *n* (%). The prevalence of the selected NCDs and risk factors are presented as *n* (%) with 95% confidence intervals (CIs). Cohen’s kappa was used to assess the reliability of self-reported data compared with clinical notes. The key variables were included in a logistic regression to establish factors associated with NCDs. All data analysis was undertaken using IBM SPSS Statistics for Windows (version 25).

## Results

### Description of the sample

A total of 199 participants completed the survey. The mean age was 34.0 (SD 10.2) years, while the median age was 31 years (IQR 25–39, minimum to maximum 27–71, *n* = 199). The mean body mass index (BMI) was 25.2 (SD 4.2); 91 (46%) were categorised as overweight and 19 (10%) were obese. The majority were white (*n* = 151, 76%), 24 (12%) were Asian, 13 (7%) mixed race, 10 (5%) black, and 1 (1%) other. A total of 80 (40%) were employed. It was found that 75 (38%) had no qualifications, 61 (31%) had GCSEs, 7 (4%) had A levels, 1 (1%) had a degree, and 54 (27%) had other qualifications. A total of 94 (47%) were homeowners or tenants, 85 (43%) lived with family and friends, 13 (7%) were rough sleepers, and 7 (4%) were in temporary accommodation.

A quarter (*n* = 48, 24%) were in prison for the first time and 58 (29%) were on remand. The median number of previous offences was 3 (IQR = 0–8, minimum to maximum = 0–100), the median expected prison stay was 8.8 months (IQR = 4.8–30.0, minimum to maximum = 1–180, *n* = 50), and the median length of sentence was 34 months (IQR = 14.8–49.5, minimum to maximum = 2–192, *n* = 138).

### Prevalence of NCDs

Table 1 shows the prevalence of NCDs: hypertension, heart disease, stroke, cancer, respiratory diseases, diabetes, and anxiety and depression. Overall, 107 (54%) reported no NCD. A total of 46% reported at least one NCD, with 60 (30%) reporting one, 26 (13%) reporting two, and 6 (3%) reporting three or more. The most common self-reported diseases were anxiety and depression (34%), respiratory disease (17%), and hypertension (10%). For any physical health NCD (excluding anxiety and depression), 147 (74%) reported no physical health NCD. A total of 26% reported at least one physical health NCD, with 42 (21%) reporting one, 8 (4%) reporting two, and 2 (1%) reporting three or more.

**Table 1.** Prevelance of NCDs

	Self-report *n* (%; 95% CI) *n* = 199	Clinical record *n* (%, 95% CI) *n* = 197	Kappa (*P* value)
Hypertension	20 (10; 6 to 14)	9 (5; 2 to 8)	0.62 (<0.001)
Heart disease	4 (2; 0 to 4)	2 (1; 0 to 2)	0.80 (<0.001)
Stroke	3 (2; 0 to 3)	2 (1; 0 to 2)	0.50 (<0.001)
Cancer	3 (2; 0 to 3)	1 (1; 0 to 2)	0.50 (<0.001)
Respiratory disease	34 (17; 12 to 22)	45 (21; 17 to 29)	0.74 (<0.001)
Diabetes	3 (2; 0 to 3)	4 (2; 0 to 4)	0.86 (<0.001)
Anxiety and depression	68 (34; 28 to 41)	61 (31; 25 to 37)	0.61 (<0.001)

CI = confidence interval. NCD = non-communicable disease.

The agreement between clinical records and self-report ranged from 0.50 (moderate) to 0.86 (very good).

Participants were asked if they had suffered from past psychiatric illness and 87 (44%, 95% CI = 37% to 51%) stated that they had, 56 (28%, 95% CI = 22% to 34%) self-harmed, and 42 (21%, 95% CI = 15% to 27%) had attempted suicide. A high proportion were receiving medication for mental health issues (*n* = 83, 42%, 95% CI = 35% to 49%).

### Prevalence of risk factors

#### Drug and alcohol dependence

A total of 53 (27%, 95% CI = 21% to 33%) self-reported a history of drug dependence, and agreement with clinical records was moderate (kappa 0.60; *P*<0.001). Thirty-one (16%, 95% CI = 10% to 21%) self-reported a history of alcohol dependence but agreement with clinical records was fair (kappa 0.38; *P*<0.001).

#### Smoking

The prevalence of smoking before imprisonment was high (*n* = 165, 83%, 95% CI = 78% to 88%), with 155 (94%) of these describing themselves as regular smokers: 75 (48%) smoked <10 cigarettes a day, 50 (32%) smoked 11–20, and 29 (19%) >20 a day (missing data = 1).

The prevalence at the time of the questionnaire was still high (*n* = 163, 82%, 95% CI = 77% to 87%). The majority (*n* = 149) continued to smoke in prison, 16 had quit in prison, 14 had started in prison and 20 remained non-smokers.

Of the 155 prisoners who smoked regularly before imprisonment, 12 (8%) quit, 38 (25%) reduced their consumption, 76 (49%) consumed the same, and 28 (18%) increased their consumption (one missing).

#### Physical exercise

Half described their level of physical activity before prison as very active (*n* = 99, 50%), 73 (37%) as fairly, 23 (12%) as not very, and 4 (2%) as not at all. In prison, the majority (*n* = 144, 72%) stated that their levels of physical activity were lower than before prison, 20 (10%) said the same, and 35 (18%) said higher.

Table 2 highlights the three most popular forms of recent (within the last month) exercise as moderate walking, weight training, and circuit training. A total of 37 (19%) took no physical activity, 44 (22%) took <150 minutes a week, and 118 (59%) took ≥150 minutes.

**Table 2.** Activity levels for type of exercise over the last month (hours)

Type of activity	Mean (SD)	Median (IQR)
Badminton and/or tennis	27.6 (12.4)	0 (0–0)
Basketball and/or volleyball	3.3 (25.7)	0 (0–0)
Circuit training	122.6 (349.3)	0 (0–0)
Moderate walking	553.1 (962.2)	100 (0–720)
Running and/or jogging	74.6 (340.5)	0 (0–0)
Football and/or hockey	15.1 (63.2)	0 (0–0)
Vigorous walking	34.4 (336.0)	0 (0–0)
Weight training	308.2 (500.2)	0 (0–0)
Other physical activity	177.2 (1038.2)	0 (0–0)
Total mean per week	329.0 (417.2)	210 (60–435)

IQR = interquartile range. SD = standard deviation.

#### Dietary intake

Regarding diet, Table 3 shows the majority eat fruit (80%) and almost half reported eating salad and/or vegetables (45%) and salt (41%) daily.

**Table 3.** Dietary intake

		*n* (%)
Salt consumption	Daily	82 (41)
	Weekly	117 (59)
	Monthly	0 (0)
	Rarely	0 (0)
Fruit	Daily	159 (80)
	Weekly	23 (12)
	Monthly	2 (1)
	Rarely	15 (7)
Salad or vegetables	Daily	89 (45)
	Weekly	72 (36)
	Monthly	7 (4)
	Rarely	31 (15)

#### Factors associated with NCDs

Demographic variables along with smoking before prison, drug dependence, alcohol dependence, weekly levels of physical activity, and dietary behaviours were included in a logistic regression analysis to explore the factors associated with NCD prevalence. Small numbers of prevalence rates precluded inclusion of each of the NCDs in the model. Therefore, just the three most common diseases (anxiety and depression, respiratory disease, and hypertension), and aggregates of 'any NCD' and 'any physical NCD' were included. Table 4 highlights that 'any NCD' was independently associated with either drug or alcohol dependence. 'Any physical NCD' was independently associated with increasing age or drug dependence. For respiratory disease, increasing age, drug dependence, alcohol dependence, fruit intake, and physical inactivity were all independently associated. For hypertension, only increasing age was independently associated. For anxiety and depression, younger age, drug dependence, alcohol dependence, fruit intake, and physical inactivity were all independently associated.

**Table 4.** Associations between demographics variables and prevalence of NCD

	Any NCD				Any physical NCD				Respiratory disease				Hypertension				Anxiety and depression			
	OR	95% CI		*P* value	OR	95% CI		*P* value	OR	95% CI		*P* value	OR	95% CI		*P* value	OR	95% CI		*P* value
		Lower	Upper			Lower	Upper			Lower	Upper			Lower	Upper			Lower	Upper	
**Age**	1.02	0.99	1.06	0.189	1.10	1.05	1.15	**<0.001**	0.95	0.91	0.99	**0.034**	1.21	1.11	1.32	**<0.001**	0.95	0.91	0.99	**0.034**
**BMI**	1.01	0.94	1.10	0.738	1.06	0.97	1.16	0.228	1.07	0.98	1.16	0.149	0.99	0.83	1.19	0.930	1.07	0.98	1.16	0.149
**Ethnic group**				0.851				0.974				0.997				0.967				0.997
White	1				1				1				1				1			
Black	0.52	0.12	2.52		1.52	0.31	7.58		0.71	0.09	5.67		0.38	0.02	5.86		0.71	0.09	5.67	
Asian	0.97	0.35	2.70		0.93	0.25	3.50		0.90	0.30	2.76		0.00	0.00			0.90	0.30	2.76	
Mixed	0.52	0.12	2.26		1.16	0.31	6.84		0.88	0.18	4.32		1.56	0.08	30.35		0.88	0.18	4.32	
Other	0.00	0.00			0.00	0.00	0.00		0.00	0.00			0.00	0.00			0.00	0.00		
**Housing status**				0.481				0.073				0.064				0.321				0.064
Temporary	1				1				1				1				1			
Rough sleeper	0.19	0.01	2.80		0.20	0.02	2.13		0.04	0.00	0.79		0.90	0.04	19.21		0.04	0.01	0.79	
Home owner or tenant	0.27	0.03	2.84		0.14	0.02	0.94		0.07	0.01	1.04		0.15	0.01	1.94		0.07	0.01	1.04	
Family or friends	0.19	0.02	1.98		0.40	0.06	2.81		0.04	0.00	0.54		0.14	0.01	2.06		0.04	0.01	0.54	
**Employed (No**)	1.21	0.58	2.51	0.610	1.13	0.47	2.76	0.784	1.30	0.57	2.97	0.538	0.95	0.17	5.43	0.954	1.30	0.57	2.97	0.538
**Smoking status before prison (Yes**)	1.51	0.61	3.76	0.377	1.45	0.47	4.45	0.521	2.43	0.77	7.65	0.129	2.52	0.25	25.14	0.430	2.43	0.77	7.654	0.129
**Drug dependence (self-report) (Yes**)	2.74	1.16	6.49	**0.022**	2.70	1.01	7.16	**0.047**	4.16	1.67	10.38	**0.002**	0.28	0.04	1.91	0.194	4.16	1.67	10.381	**0.002**
**Alcohol dependence (self-report) (Yes**)	3.90	1.32	11.51	**0.014**	1.67	0.58	4.76	0.344	4.63	1.60	13.42	**0.005**	1.70	0.26	11.07	0.578	4.63	1.60	13.42	**0.005**
**Physical activity**				0.212				0.040				**0.048**				0.455				**0.048**
None	1				1				1				1				1			
<150 minutes	0.39	0.13	1.20		0.21	0.05	0.91		0.30	0.09	1.04		0.74	0.05	11.44		0.30	0.09	1.04	
≥150 minutes	0.47	0.18	1.20		1.05	0.37	2.94		0.27	0.09	0.77		2.63	0.34	20.39		0.27	0.09	0.77	
**Fruit**				0.121				0.117				0.049				0.467				0.049
Daily	1				1				1				1				1			
Weekly	1.51	0.51	4.45		0.40	0.09	1.85		2.85	0.89	9.09		0.00	0.00			2.85	0.89	9.09	
Monthly	1.10	0.04	31.17		20.40	0.49	854.59		1.69	0.03	87.74		158.97	0.32	80293.06		1.69	0.03	87.74	
Rarely	0.19	0.04	0.86		0.29	0.05	1.82		0.12	0.01	0.94		0.00	0.00			0.12	0.01	0.94	
**Salad or vegetables**				0.260				0.396				0.793				0.591				0.793
Daily	1				1				1				1				1			
Weekly	1.44	0.65	3.18		1.39	0.55	3.51		1.07	0.44	2.60		2.51	0.52	12.25		1.07	0.44	2.60	
Monthly	0.53	0.07	3.94		0.63	0.03	13.66		0.36	0.04	3.19		3.98	0.02	706.59		0.36	0.04	3.19	
Rarely	2.58	0.87	7.67		2.74	0.80	9.43		0.86	0.26	2.82		0.57	0.02	20.89		0.86	0.26	2.82	
**Salt consumption**				0.869				0.343				0.579				0.822				0.579
Daily	1				1				1				1				1			
Weekly	1.06	0.54	2.08	0.869	1.47	0.67	3.23		0.81	0.39	1.70	0.579	1.18	0.28	4.93	0.822	0.81	0.39	1.70	0.579

BMI = body mass index. BP = blood pressure. CI = confidence interval. NCD = non-communicable disease.

## Discussion

### Summary

This research shows that, compared with mainstream populations, with the exception of respiratory disease, physical illness NCDs are much less common.^22^ However, poor mental health is more common. The prevalence statistics for mainstream populations are crude and unadjusted for demographic variables. It is therefore possible that demographic factors, in addition to socioeconomic determinants, account for the difference in prevalence between prison and mainstream populations. For example, the median age of participants (at 31 years) would suggest a lower median age than the mainstream UK population (40 years).^23^ These findings, that approximately 38% of the sample had no educational qualifications and that 60% were unemployed before imprisonment, would also suggest a lower educational status and lower employment status than the mainstream UK population.^24,25^

Prevalence of any NCD was associated with a history of drug or alcohol dependence. Prevalence of a physical NCD was associated with increasing age, whereas prevalence of anxiety and depression was independently associated with either younger age or not taking physical exercise. Prevalence of risk factors for NCDs were high, with poor vegetable intake (although prevalence of daily fruit intake reported high at 80%), use of added salt, high tobacco consumption, and lower physical activity compared to pre-imprisonment levels reported. While some prisoners exercise well above the recommended minimum level, some do no or very little exercise. However, despite reported lower levels of physical exercise in the prison setting and restricted food choices, compared with community populations, participants had lower levels of obesity (mean BMI was 25 for the sample versus 27.3 for community populations).^10^

Self-report by prisoners shows moderate to very good reliability for common disorders such as drug dependence, respiratory disease, and anxiety and depression. The kappa value for alcohol dependence, while statistically significant, is only fair, indicating prisoners may, on reception into prison, report a history of alcohol dependence to clinicians for secondary gain (for example, obtaining benzodiazepine medication for subsequent diversion in the shadow prison economy).

### Strengths and limitations

This study is the first UK multi-centre prevalence study of NCDs in prisons, and the first European study that the authors are aware of employing such data-linkage methodology.

This research is limited in that, while 95% of the UK prisoner population is male,^2^ data were not collected from either female prisoners or prisoners resident in high-secure settings, thus limiting generalisability.

### Comparison with existing literature

The reported prevalence of 82% smokers among the prison population would suggest a higher prevalence than community populations, which is reported as 15.5% among adults in England.^26^ Such a high prevalence also explains why an independent association between prevalence and type of NCD was not found, since prevalence was so high across all groups.

The reported statistics of 45% consuming salad or vegetables each day and 80% consuming fruit each day cannot be directly compared with reported community rates. The Health Survey for England 2013 found fruit or vegetable consumption among males in the general population to be on average 3.5 portions per day, with 25% consuming five or more portions.^27^ Such findings would suggest a lower consumption of fruit and vegetables among prisoner populations, although more research is needed to verify the preliminary findings.

The research also showed that over one-third of participants either took no physical exercise or took less than the UK Department of Health (DoH) recommended level of ≥150 minutes/week of aerobic moderate physical activity (MPA), or 75 minutes/week of vigorous physical activity (VPA) (or an equivalent combination), in bouts of 10 minutes or more. The majority of participants reported a reduction in physical activity levels on reception into prison and such findings concur with a study of UK drug-using prisoners in which aerobic physical activity reduced by a factor of >2.5 following reception into prison.^28^

However, in this study, 59% met or exceeded DoH minimum levels pertaining to exercise, with some greatly exceeding these levels, principally through either moderate walking or weight training. The 2012 Health Survey for England found that among the general population, 67% of men met the DoH recommended level of exercise and just 19% of men were classed as inactive (defined as <30 minutes/week of MPA or 15 minutes/week of VPA, or an equivalent combination of these).^29^ Therefore, these findings would suggest similar levels of physical inactivity among male prisoners and the general population.

In 2015, in UK community populations, 68% of men were overweight or obese, and obesity prevalence had increased from 15% in 1993 to 27% in 2015.^30^ In the present study, 46% were categorised as overweight and 10% were categorised as obese. A mean BMI of 25 would suggest that UK prisoner populations are not clinically underweight. However, this result should be interpreted with caution since anabolic steroid use is common among male prisoner populations with an explicit intent of building muscle body mass.^31,32^

Regarding data-linkage studies between surveys and clinical records, the authors are only aware of one previous such prison-based study in which comparative statistics for agreement were reported. Conducted in the US among 679 inmates from one male and one female maximum-security prison, findings concurred with the findings from the present study; namely, good to very good agreement for diabetes, asthma, and hypertension.^33^ While the study outcomes highlighted a poor agreement for 'illicit drug use ever', the present study found moderate agreement for the variable of 'drug dependence' and evidence of this in their medical record.

### Implications for research and practice

This research would suggest targeted behaviour change interventions for prisoners, while acknowledging dietary modification is not routinely possible in prisons since menus are predetermined.^34^ There is limited empirical research to inform the content or medium of delivery of such prison-specific interventions, or indeed whether such interventions should be professionally-led or peer-led. There is some evidence to suggest the effectiveness of peer-led interventions in addressing risk factors for either HIV or drug dependence in prisoner populations, and it is suggested that the application of such interventions to address risk factors for NCDs is an area that merits further research activity.^35,36^

With the exception of conditions where there is potential secondary gain (for example, stating a diagnosis of alcohol dependence to prison clinicians in an effort to secure medication that has potential for abuse), this research would suggest high level of agreement between prisoner self-report and medical records. Therefore, it is suggested that medical records are a valid database to inform prevalence statistics necessary to inform future policy and practice.

The research has highlighted the high prevalence of risk factors for NCDs, despite below mainstream population mean prevalence of such conditions, which is likely explained by the young mean age of prisoner populations. This presents a window of opportunity for intervention. Since 2004, an incentive scheme in community primary care practice, the Quality and Outcomes Framework (QOF), provides financial remuneration to GPs who achieve standards in 'clinical', 'organisational', 'additional services', and 'patient experience' domains.^37^ The clinical domain initially contained standards for 10 chronic conditions, rising to 19 in the financial year 2017/18.^38,39^ However, there is no contractual obligation placed on prison-based GPs to undertake monitoring of QOF clinical indicators. Arguably, this presents a missed opportunity to raise prison-based clinical standards. The research would suggest benefit from QOF monitoring through the commissioner-led clinical service specifications. Further, achieving QOF indicators should attract financial remuneration, thus increasing the likelihood of management of NCDs being of an equivalent standard to that provided in community settings.
